# Prediction of the Wall Factor of Arbitrary Particle Settling through Various Fluid Media in a Cylindrical Tube Using Artificial Intelligence

**DOI:** 10.1155/2014/438782

**Published:** 2014-03-18

**Authors:** Mingzhong Li, Guodong Zhang, Jianquan Xue, Yanchao Li, Shukai Tang

**Affiliations:** ^1^College of Petroleum Engineering, China University of Petroleum, B405 Engineering Building, No. 66 Changjiang West Road, Qingdao 266580, China; ^2^Down Hole Company, Chuanqing Drilling Company, CNPC, Chengdu 610051, China; ^3^Dongsheng Group Co., Ltd. of Shengli Oilfield, Dongying 257000, China

## Abstract

Considering the influence of particle shape and the rheological properties of fluid, two artificial intelligence methods (Artificial Neural Network and Support Vector Machine) were used to predict the wall factor which is widely introduced to deduce the net hydrodynamic drag force of confining boundaries on settling particles. 513 data points were culled from the experimental data of previous studies, which were divided into training set and test set. Particles with various shapes were divided into three kinds: sphere, cylinder, and rectangular prism; feature parameters of each kind of particle were extracted; prediction models of sphere and cylinder using artificial neural network were established. Due to the little number of rectangular prism sample, support vector machine was used to predict the wall factor, which is more suitable for addressing the problem of small samples. The characteristic dimension was presented to describe the shape and size of the diverse particles and a comprehensive prediction model of particles with arbitrary shapes was established to cover all types of conditions. Comparisons were conducted between the predicted values and the experimental results.

## 1. Introduction

Walls exert an extra retardation on particle settling in diverse fluid media, which can lower the terminal settling velocity of particles due to the upward flow of fluid in the narrow gaps between the particle and the wall. Knowledge of the terminal settling velocity of particles in bounded fluid is of great importance in various industrial applications such as process engineering, three-phase fluidized bed reactors, separation in multiphase systems, membrane transport, and hydraulic transport systems. To determine the net hydrodynamic drag force on a particle, it is common to introduce a wall factor, *F*_*w*_, to quantify the retarding effect of the wall on the falling particles.

Consider the following:

(1) $$\begin{array}{c} \;F_{w}=\frac{V}{V_{\infty }}, \end{array}$$

where *V* and *V*_*∞*_ are the terminal velocity of a particle in bounded and unbounded fluids.

There are numerous studies investigating the wall effect on different-shaped particles settling in Newtonian and non-Newtonian fluids. Some researchers measured the terminal settling velocity of spheres in Newtonian fluids and found that the wall factor is a function of the sphere-to-tube diameter ratio, *λ* = *d*/*D*, in different Reynolds number regions [1–4]. The wall factor for spheres settling in Newtonian fluids is dependent only on the *λ* at both the viscous and inertial regions, whereas at the intermediate transition regime, the wall factor is a function of both the *λ* and the Reynolds number. Chhabra, et al. culled 1260 data points from the literature that cover a wide range and assessed the validity of many known wall correction formulates [5]. The results show that the Haberman and Sayre theoretical expression (2) and Newton expression (3) are the most reliable in viscous and turbulent regions, respectively [6, 7], whereas (4a) and (4b), given by di Felice, are recommended in the intermediate regime [8].

Consider the following:

(2)   Fw=1−2.105λ+2.0865λ3−1.7068λ5+0.72603λ61−0.75857λ5,

(3)   Fw=(1−λ2)(1−0.5λ2)0.5,

(4a) $$\begin{array}{c} F_{w}={\left(\frac{1-\lambda }{1-0.33\lambda }\right)}^{\alpha }, \end{array}$$

where *α* is related to the terminal Reynolds number as

(4b) 3.3−αα−0.85=0.1Re,

where Re is the terminal Reynolds number of sphere.

Chhabra and Uhlherr [9] and Lali et al. [10] presented the wall effect on the settling velocity of spheres in non-Newtonian solutions at high Reynolds numbers (1.0 ≤ *Re* ≤ 10^3^) and low Reynolds numbers (2 × 10^−3^ ≤ *Re* ≤ 150) separately, and corresponding correlations (5) and (7) were established. Machač and Lecjaks [11] and Malhotra and Sharma [12] established wall factor correlations for spheres settling through power law fluids and surfactant-based shear thinning viscoelastic fluids in rectangular ducts and parallel plates. Kawase and Ulbrecht [13], Missirlis et al. [14], and Song et al. [15] theoretically studied and numerically simulated the settling velocity of a sphere in bound non-Newtonian fluid. It is now widely accepted that the elasticity and shear thinning behavior of non-Newtonian fluids reduce the retardation effect of the confining walls.

Consider the following:

(5) φ−φ∞φ0−φ∞=[1+(ARe)2]−B,

(6) Re=dnV2−nρK,

where *φ* is the reciprocal of the wall factor *F*_*w*_. and *φ*_0_ and *φ*_*∞*_ are the asymptotic values of *φ* at low and high Reynolds numbers, respectively. *A* and *B* are two curve fitting parameters. *d* is sphere diameter. *ρ* is fluid density. *K* is consistency index of fluid media.

Consider the following:

(7) $$\begin{array}{c} F_{w}={\left(1-\lambda \right)}^{\left(a+n(n-1)\right)/\left(1+b(n-1)\right)}{\left(\text{R}{\text{e}}_{\infty }\right)}^{b/\left(1+b(n-1)\right)}, \end{array}$$

where *a* = 1.8 and *b* = 0.1.  *n* is flow behavior index.

Extensive research is available on the settling velocity of nonspherical particles in various fluid media. Unnikrishnan and Chhabra [16, 17] studied the settling behavior of numerous cylinders in Newtonian and non-Newtonian fluids, discussed the wall effect in a manner analogous to those for spherical particles, and analyzed the terminal velocity using a drag coefficient-Reynolds number approach. The settling velocities of cylinders, needles, and rectangular prisms in shear thinning polymer solutions were measured by Madhav and Chhabra [18]. Chhabra [19, 20] investigated the effect of confining boundaries on the terminal settling velocity of cylinders, rectangular prisms, needles, thin plates, circular discs, and prisms in viscous media and non-Newtonian polymer solutions and quantified the retardation effect in terms of the wall factor (8).

Consider the following:

(8) Fw=f(Re,dD,shape).

To the best of our knowledge, the wall factor correlations vary with the particle shape, the fluid properties, and the flow regimes, which are divided into viscous flow, inertial flow, and intermediate Reynolds number regions. The wall effect on the settling velocity of cylinders that are classified as short cylinders (*L*/*d* < 10) and slender cylinders (*L*/*d* > 10) was quantified using two expressions by Chhabra [19, 20]. Therefore, a reliable model that can be used to quantify the wall effect on the settling velocity of particles with arbitrary shapes in various fluid media in all types of conditions is not yet available. The objectives of this study are as follows: (a) to classify particles of various shapes into three types: sphere, cylinder, and rectangular prism and establish a prediction model of the wall factor for each type of particle using an artificial neural network and support vector machine; (b) to present a characteristic dimension that describes the size and shape of arbitrary particles and provide a comprehensive prediction model for arbitrary particles in all types of conditions.

## 2. Database

**Figure 1:**
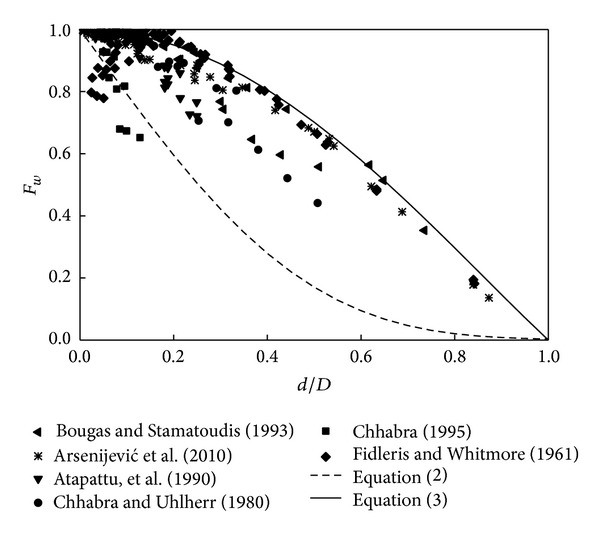
Dependence of wall factors for spheres on the sphere-tube diameter ratio.

To cover a wide range of conditions, the experimental data of differently shaped particles settling in various fluid media were culled from ten papers, yielding a total of 513 data points. The number of spheres is 216, as shown in Figure 1; cylinders and rectangular prisms make up 251 and 46 data points, respectively. No precise data are available because all of the data were presented in graphical form. Many other investigators, for example, Delidis and Stamatoudis [3], Lali, et al. [10], and Strnadel et al. [22], presented numerous experimental results on boundary effects, but sufficient details have not been reported, such as the density of the fluid or the particle and the rheological parameters of liquid. Uhlherr and Chhabra [23] and Chhabra et al. [24] provided their results in the form of *F*_*w*_-Re relationship, which is also inadequate for this paper. Fidleris and Whitmore [1] measured the terminal settling velocity of steel spheres falling axially in cylindrical vessels through water. To obtain the wall factors, (9) was used to calculate the falling velocity of the spheres in infinite fluid, which was validated to have an excellent degree of fit with 480 experimental data points culled from 16 papers by Brown and Lawler [25] at all Reynolds numbers less than 2 × 10^5^. Research on rectangular prisms is rare, which led to minimal related data. A summary of the culled data with other details is provided in Table 1.

Consider the following:

(9) u∗=[(18d∗2)((0.936d∗+1)/(d∗+1))0.898+(0.317d∗)0.449]−1.114,

(10)   d∗=d[gρ(ρp−ρ)μ2]1/3,

where *u*_∗_ is dimensionless settling velocity. *d*_∗_ is dimensionless sphere diameter. *μ* is absolute fluid viscosity.

Brown and Lawler [25] used 5 parameters, (11)–(14), to evaluate the correlations of sphere drag, which were also available in this paper. The sum of the squared errors, *Q*, is defined in (11).

Consider the following:

(11) $$\begin{array}{c} Q=\sum_{1}^{k}{\left(\log F_{w\exp}-\log F_{w\;\text{pre}}\right)}^{2}, \end{array}$$

where *F*_*w*exp⁡_, *F*_*w* pre_ are experimental wall factor and predicted wall factor, respectively.

The indication of the average displacement of predicted *F*_*w*_ from the experimental results can be determined by the root-mean-square deviation, as shown in (12).

Consider the following:

(12) $$\begin{array}{c} \text{de}{\text{v}}_{\text{rms}}=\sqrt{\frac{Q}{k}}. \end{array}$$

The sum of the relative errors and the sum of the squares of the relative errors are two different parameters for the goodness of fit and are defined in (13).

Consider the following:

(13) $$\begin{array}{c} \delta =\sum_{1}^{k}\frac{\left|F_{w\exp}-F_{w\;\text{pre}}\right|}{F_{w\exp}},\\ Q_{\mathrm{rel}}={\sum_{1}^{k}\left(\frac{F_{w\exp}-F_{w\;\text{pre}}}{F_{w\exp}}\right)}^{2}. \end{array}$$

The correlation coefficient is shown in (14).

Consider the following:

(14) $$\begin{array}{c} \gamma =\frac{\sum F_{w\exp}F_{w\;\text{pre}}-\sum F_{w\exp}\sum F_{w\;\text{pre}}/k}{\sqrt{\left(\sum F_{w\exp}^{2}-{\left(\sum F_{w\exp}\right)}^{2}/k\right)\left(\sum F_{w\;\text{pre}}^{2}-{\left(\sum F_{w\;\text{pre}}\right)}^{2}/k\right)}} \end{array}$$

## 3. Theory

### 3.1. Feature Parameters

Feature parameters are the input variables of ANN and SVM and the dependence of prediction accuracy of model. The extraction of feature parameters should follow three principles: (a) they should be easy to obtain; (b) significantly affect the outputs; and (c) be uncorrelated. The expression of the terminal settling velocity of spheres in infinite non-Newtonian fluid presented by Novotny [26] is shown in (15). The settling velocity is a function of the density of the fluid and particles (*ρ*, *ρ*_*p*_), the fluid rheological parameters (*K*, *n*), and the sphere diameter (*d*). In addition, the tube-to-sphere diameter ratio (*λ*) can represent the retardation effect of the wall; therefore, *ρ*, *ρ*_*p*_, *d*, *λ*, *K*, and *n* were chosen as feature parameters of spheres. Cylinders, plates, needles, and discs have similar shapes, and their size can be determined by diameter (*d*) and length (*L*). Therefore, these four types of particles can be treated as one type. The density of the particles and the fluid, *d*, *d*/*D*,  *d*/*L*, the consistency index (*K*), and the flow behavior index (*n*) were selected as inputs (*ρ*, *ρ*_*p*_, *d*, *d*/*D*, *d*/*L*, *K*and  *n*) of the ANN model. A specific rectangular prism can be defined by the length (*l*), width (*w*), and height (*h*), so *ρ*, *ρ*_*p*_, *l*, *w*, *h*, *D*, *K*, and *n* were selected as inputs of the SVM prediction model for rectangular prisms.

Consider the following:

(15) $$\begin{array}{c} V_{\infty }={\left[\frac{g\left(\rho -\rho_{p}\right)d}{18K}\right]}^{1/n}d. \end{array}$$

The maximum particle density was 8900 kg/m^3^, and none of the sphere diameters exceeded 32 mm and they were not on the same scale. To avoid the negative effect of oversize and undersize, the input data were normalized to a specific range by transformation processes. Equation (16) was used to transform the data of the input into the interval of [−1, 1].

Consider the following:

(16) $$\begin{array}{c} f=2\frac{y-y_{\min}}{y_{\max}-y_{\min}}-1, \end{array}$$

where *f* is the normalized parameters, and *y*_*w*min⁡_ and *y*_*w*max⁡_ are the minimum and maximum of the actual data, respectively.

### 3.2. Artificial Neural Network

An artificial neural network is a machine-learning algorithm that attempts to mimic the acquisition and organization skills of biological neural networks based on the empirical risk minimization (ERM) principle. It has been widely used in various engineering fields due to its ability to solve complex and nonlinear relationships. It offers important support in two aspects: pattern recognition [27–30] and prediction [31–34]. No works have been performed on the prediction of the wall factors of particles settling in finite fluids using an ANN except for the works of Rooki et al. [35] and Ghamari et al. [36], who used ANNs to relate the settling velocity with solid spheres and seeds.

Back-propagation neural network (BPNN) has obtained an increasing popularity in control analysis, prediction analysis, pattern recognition, and fault diagnosis of mechanical equipment with error back propagation. Werbos [37] proposed the BP learning theory in 1974, which was improved and applied to artificial neural networks by McClelland and Rumelhart [38].

Feed-forward neural networks with back propagation (BP) of one hidden layer can map any nonlinear relationship. To reduce training time, one hidden layer was selected in this paper, and a three-layer BP neural network was constructed to predict the wall factors. The appropriate number of nodes in a hidden layer is usually determined by the empirical correlation shown in (17).

Consider the following:

(17) $$\begin{array}{c} m=\sqrt{p+q}+c, \end{array}$$

where *m* is the number of nodes in a hidden layer, *p* and *q* are the input and output nodes, respectively, and *c* is a constant in the range of 1 to 10. The ANN toolbox of MATLAB was used to implement the automated Bayesian regularization for training the BP neural network.

### 3.3. Support Vector Machine

Cortes and Vapnik [39] proposed a support vector machine (SVM) based on the VC dimension of statistical learning theory and the structure risk minimization (SRM) principle in 1995. A support vector machine is superior to artificial neural networks for resolving the problem of small samples and over-fitting, and it seeks an optimum solution for a whole situation and has a stronger generalization ability. Among the total 46 data sets of rectangular prism, 34 were selected as training data, and the other 12 were used for testing purposes. An artificial neural network was used to predict the wall factors of rectangular prisms, but the goodness of fit was poor, and different training functions and parameters were adjusted in numerous trials; unfortunately all of the efforts failed. Therefore, the support vector machine was chosen to predict the wall factors of rectangular prisms due to its superiority in dealing with small sample problems compared to the artificial neural network.

Given a training data set, (*x*_1_, *y*_2_),…, (*x*_*N*_, *y*_*N*_), where *x*_*i*_ ∈ *X*, *y*_*i*_ ∈ *R*, *N* is the size of training data set, and *X* denotes the space of the input samples; for instance, *R* ^*n*^, the decision function implemented by support vector machine can be written as

(18) $$\begin{array}{c} f\left(x\right)=\sum_{i=1}^{N}\left({\vec{\alpha }}_{i}^{*}-{\vec{\alpha }}_{i}\right)K\left({\vec{x}}_{i},\vec{x}\right)+\zeta , \end{array}$$

where $K\left({\vec{x}}_{i},\vec{x}\right)$ is the kernel function, and $\zeta \in R,\;\;{\vec{\alpha }}_{i}$ and ${\vec{\alpha }}_{i}^{*}$can be obtained by solving the follow convex Quadratic Programming (QP) problem.

Consider the following:

(19) min⁡α∗∈R2N 12∑i,j=1N(αi∗−αi)(αi∗−αj)K(xi,xj)  +ε∑i=1N(αi∗+αi)−∑i=1Nyi(αi∗+αi)

subject to

(20) $$\begin{array}{c} \sum_{i=1}^{N}\left(\alpha_{i}-\alpha_{i}^{*}\right)=0, \end{array}$$

(21) $$\begin{array}{c} 0\leq \alpha_{i},\;\alpha_{i}^{*}\leq \frac{C}{N}\;\left(i=1,2,\ldots ,N\right), \end{array}$$

where *α*_*i*_ and *α*_i_* are the corresponding Lagrange multipliers and *C* is the penalty parameter. Here, the radial basis function [RBF, (22)] was adopted.

Consider the following:

(22) $$\begin{array}{c} K\left({\vec{x}}_{i},{\vec{x}}_{j}\right)=\exp\left(-r{||{\vec{x}}_{i}-{\vec{x}}_{j}||}^{2}\right). \end{array}$$

Solving the QP problem of (19) with the constraints of (20) and (21), the optimum solution of $\vec{\overset{-}{\alpha }}=\left({\overset{-}{\alpha }}_{1},{\overset{-}{\alpha }}_{1}^{*},\ldots ,{\overset{-}{\alpha }}_{N},{\overset{-}{\alpha }}_{N}^{*}\right)$ was obtained. Therefore, the constant of *ζ* in (18) can be computed as follows:

(23) ζ=yj−∑i=1N(α−i∗−α−i)(xi·xj)+ε,   for  α−j∈(0,CN),ζ=yk−∑i=1N(α−i∗−α−i)(xi·xk)−ε, for  α−k∗∈(0,CN).

The LIBSVM toolbox (http://www.csie.ntu.edu.tw/~cjlin/libsvm/index.html) established by Chang and Lin [40] of National Taiwan University was used.

## 4. Prediction of Wall Factor

### 4.1. Sphere

An ANN model of one input layer with six inputs (*ρ*, *ρ*_*p*_, *d*, *λ*, *K*, and *n*), one hidden layer of 12 neurons, and one output layer was established. Fletcher and Goss [41] suggested that each neuron has a bias and is fully connected to all inputs and that an activation function of sigmoid hyperbolic tangent (tansig) was recommended. The linear activation function of the output layer and the automated Bayesian regularization algorithm (trainbr) of the training function were selected. Seventy-five percent of the 216 datasets were chosen as training data, and the other 54 were chosen as testing data.

**Figure 2:**
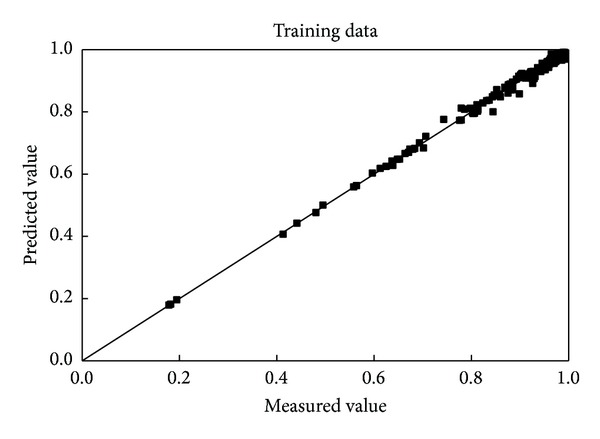
Comparison of the predicted and measured wall factor of the spheres for the training data.

Figure 2 shows that the predicted wall factor fits well with the measured data for the training data set. The correlation coefficient is 0.9975, giving a perfect fit, with 99.38% (in Table 2) of the data lying within ±5% of the measured results, which was expected because this data set was used for the training of the network. The excellent degree of fit indicates that the training was successful.

**Figure 3:**
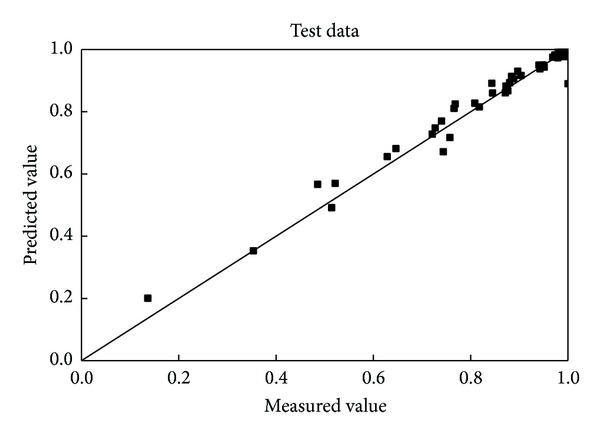
Comparison of the predicted and measured wall factors of spheres for the test data.

The test data sets were used to validate the network established above. The contrast of the predicted wall factors with the measured values for the test data set is shown in Figure 3. It is 92.59%, which means that the predicted data lies within ±10% of the measured values. There is a good engineering accuracy although the degree of fit is not as good as the training data set.

### 4.2. Cylinder

**Figure 4:**
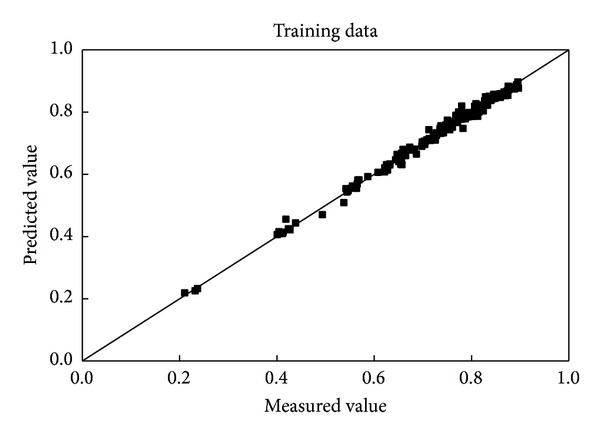
Comparison of the predicted and measured wall factors of cylinders for the training data.

**Figure 5:**
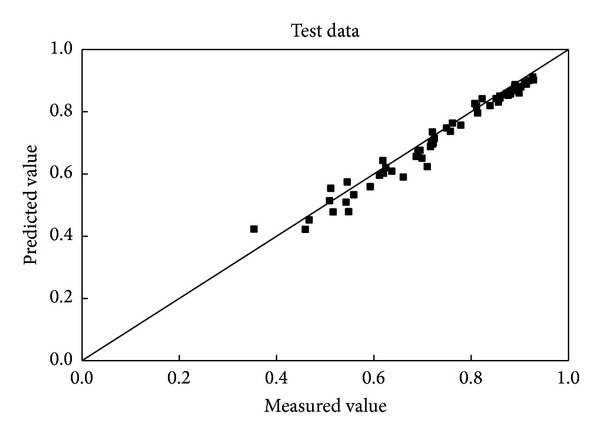
Comparison of the predicted and measured wall factors of cylinders for the test data.

Cylinders, plates, needles, and discs can be treated as one type of particle due to their similar shapes. An ANN model of seven inputs (*ρ*, *ρ*_*p*_, *d*, *d*/*D*, *d*/*L*, *K*, and *n*) was established with training and an activation function the same as those of spheres. Sixty-two data points out of 251 were used as a test set; the comparison of the predicted data with the measured results is shown in Figures 4 and 5.

The results are similar to those of the spheres. The goodness of the fit for both the training data and the test set is excellent. As shown in Table 2, the percentage of predicted values within ±7.5% of the measured data is greater than 90%, and the correlation coefficients are 0.9971 and 0.9865 for the training dataset and the test dataset, respectively.

The close fit between the predicted results and the measured values for cylinders, plates, and needles demonstrates that the wall factors of various particles with similar shapes can be predicted with the same model of ANN, which is not feasible for any correlations. Therefore, it will be significant to establish a comprehensive prediction model for arbitrary particles using ANN, which will be presented subsequently.

### 4.3. Rectangular Prism

An SVM prediction model with 8 inputs (*ρ*, *ρ*_*p*_, *l*, *w*, *h*, *D*, *K*, and *n*) was established. The number of training datasets is 34 with the other 12 data points as the test set, and all of the input and output variables were generalized by (16). The optimum *C* and *r* in (21) and (22) were determined by Grid Search Method.

**Figure 6:**
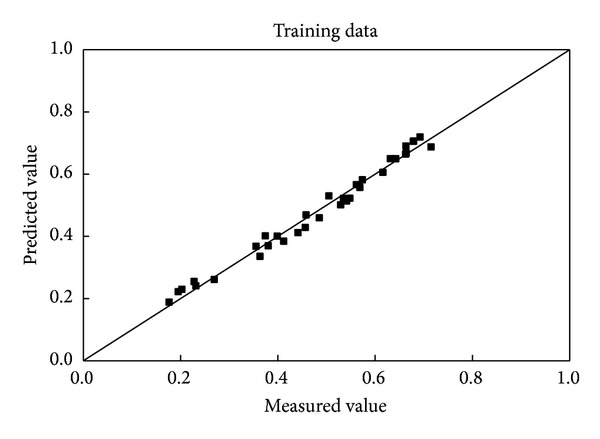
Comparison of the predicted and measured wall factors of rectangular prisms for the training data.

**Figure 7:**
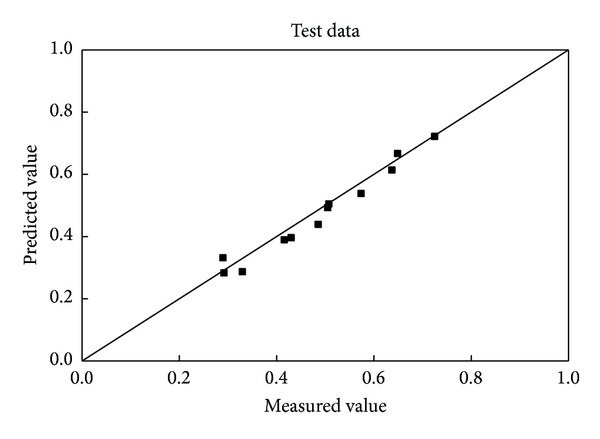
Comparison of the predicted and measured wall factors of rectangular prisms for the test data.

The contrast of the predicted values and the measured results is shown in Figures 6 and 7. As the data points are rare, the degree of fit is not as strong as with the sphere and cylinder for training datasets, but it still maintained adequate engineering accuracy. The percentages of the predicted results lying within ±10% and ±15% of the measured data were 83.33% and 91.67%, for the test data, respectively.

### 4.4. All Particles

The prediction of wall factors for differently shaped particles on settling in various fluid media was conducted by artificial intelligence. The degree of fit was not perfect for the test data set compared to the training data set, but it still maintained adequate engineering accuracy. As stated in Section 4.2, a comprehensive model that predicts the wall factor of arbitrary particles covering all types of conditions may be feasible, and it will be of crucial significance, therefore a new BP neural network was established to conduct this work in this segment.

It is difficult to describe particles with various shapes with one variable. Various works have been performed to study the effects of walls on the terminal settling velocity of nonspherical particles. The equivalent diameter was used [3, 18–20]; however, it may lack fidelity for discs and needles, the length-to-diameter ratios of which are too small or too large. The size of a sphere can be determined by the diameter, a cylinder can be defined by the diameter and length (*L*), and length (*l*), width (*w*), and height (*h*) are the three dimensions of rectangular prisms. Therefore, a characteristic dimension, defined as (*dx*, *dy*, *dz*), was used to describe the size and shape of various particles, and the characteristic dimensions for spheres, cylinders, and rectangular prisms are (*d*, 0, 0), (*d*, 0, *L*), and (*l*, *w*, *h*), respectively. A BP neural network with 8 nodes (*ρ*, *ρ*_*p*_, *dx*, *dy*, *dz*, *D*, *K*, and  *n*) for the input layer and one hidden layer with 10 nodes was established. The activation function of the input layer and the output layer used in MATLAB ANN toolbox were tansig and logsig, and the automated Bayesian regularization algorithm (trainbr) of the training function was chosen. Of the 513 data points, 128 were chosen as the test data.

**Figure 8:**
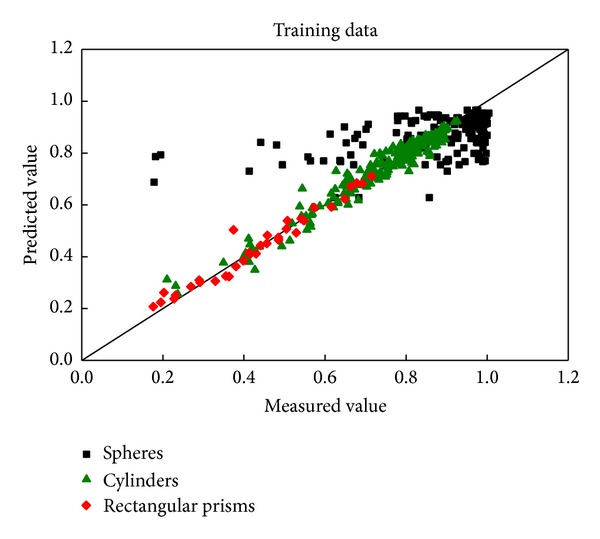
Comparison of the predicted and measured wall factors of all particles for the training data.

**Figure 9:**
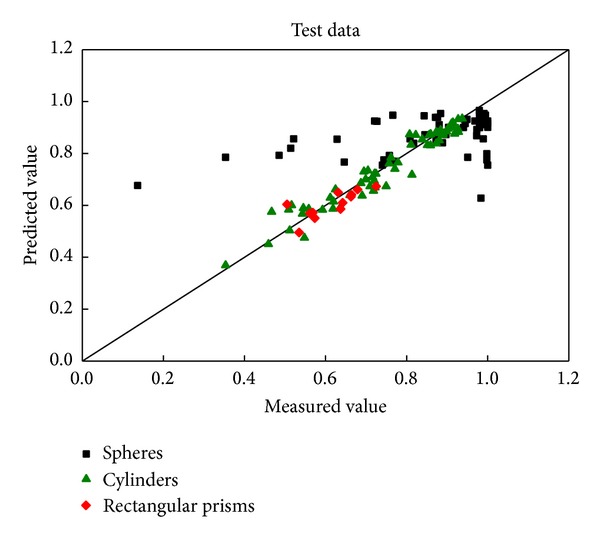
Comparison of the predicted and measured wall factors of all particles for the test data.

Figures 8 and 9 show that the predicted values of the wall factors for cylinders and rectangular prisms fit well with the experimental results. A total of 95.24% and 90.32% of the predicted data for the cylinders were within ±10% of the measured results for training set and test set, respectively. These values were 85.29% and 91.67% for rectangular prisms. Compared with the single model; the predicted results of rectangular prisms are greater for the test set than the training set, which may due to the increase of sample number.

The contrast between the predicted values and the experimental results of spheres is significant. The goodness of fit for the predicted values to the measured data is shown in Table 3. More than 30% of the predicted values are beyond ±10% of the measured data, which cannot meet the requirement of engineering accuracy. The probable reason is that the characteristic dimension is not so appropriate. Compared to cylinders and rectangular prisms, the dimensions of spheres are described only by the diameter with the other two artificial variables set as zero, (*d*, 0,0), which maybe the primary reason for this problem.

To improve the prediction accuracy and eliminate the problem mentioned above, two main measures should be taken: (1) establishment of a more reliable parameter to accurately represent the size and shape of an arbitrary particle and (2) classification of all particles into two types, spherical particles and nonspherical particles and construction of the prediction model using artificial intelligence.

## 5. Conclusion

Previous correlations of wall factor can only be applied to particles with specific shapes falling in fluid media with specific properties, whether Newtonian or non-Newtonian fluids. Two new prediction methods for wall factors for the settling of arbitrary-shaped particles through various fluid media using artificial intelligence have been presented. A data set of 513 points was produced from experimental data from ten studies, and the numbers of spheres, cylinders, and rectangular prism were 216, 251, and 46, respectively. Due to the small number of samples, a support vector machine was used to predict the wall factors of rectangular prisms because it is more compatible for small sample sets. Finally, characteristic dimensions were presented to describe the shape and size of diverse particles, and a new BPNN prediction model of wall factors for arbitrary-shaped particles covering all types of conditions was established.

Comparisons were conducted between the predicted data and the experimental values. The degree of fit of the training dataset was superior to that of the test set for the single model, and both achieved adequate engineering accuracy. The predicted values lie within ±10% of the measured data except for the test data of rectangular prisms because of the sample set. The goodness of fit of the comprehensive prediction model for arbitrary-shaped particles varied greatly with particle shape, which is greatly affected by the characteristic dimensions of the particles, and a more reliable parameter is needed to represent size and shape of arbitrary-shaped particles.

## Figures and Tables

**Table 1:** Range of conditions covered by the data.

Source	Particle shape	Range of Re	Range of fluid properties		Number of data points
			*K*	*n*	
Chhabra and Uhlherr [9]	Sphere	1.52~13.92	0.123	0.8	29
Chhabra [19]	Sphere, cylinder, plate, needle, disc and cube	<7	0.548~0.91	1	116
Arsenijević et al. [4]	Sphere	53~15100	0.001	1	43
Fidleris and Whitmore [1]	Sphere	1971~19205	0.001	1	58
Atapattu et al. [21]	Sphere	0.002~0.182	3.69~9.3	0.43~0.53	44
Bougas and Stamatoudis [2]	Sphere	13500~77000	0.001	1	30
Chhabra [20]	Sphere, cylinder, plate, needle, disc and cube	<7	0.464, 0.741	0.779, 0.79	66
Madhav and Chhabra [18]	Cylinder, needle and rectangular prism	0.05~150	0.092	0.965	55
Unnikrishnan and Chhabra [16]	Cylinder	3.8 × 10^−4^~1.27	1.89, 10.53	0.62	40
Unnikrishnan and Chhabra [17]	Cylinder	0.2~180	0.406, 0.49	1	32

**Table 2:** Fit of experimental results to the predicted values of a single model.

Correlated	Sum of squared error (*Q*)	rms^a^ deviation (dev_rms_)	Sum of squared relative error (*Q*_*rel*⁡_)	Sum of relative error (*δ*)	Correlation coefficient (*γ*)	Range analysis: predicted data points within specified range of experimental results			
						±15%	±10%	±7.5%	±5%
Sphere (train)	0.0051	0.0056	0.0271	1.5262	0.9975	100	100	100	99.38
Sphere (test)	0.0537	0.0315	0.3738	2.0232	0.971	94.45	92.59	88.89	79.63
Cylinder (train)	0.0099	0.0072	0.0529	2.1864	0.9971	100	100	99.47	98.41
Cylinder (test)	0.0272	0.0209	0.1432	2.1089	0.9865	98.39	93.55	90.32	82.26
Rectangular prism (train)	0.0197	0.0241	0.1098	1.56	0.9914	100	91.18	88.24	61.77
Rectangular prism (test)	0.0089	0.0272	0.048	0.5687	0.9922	91.67	83.33	66.67	50

^a^rms indicates root-mean square.

**Table 3:** Fit of the experimental results to the predicted values of the comprehensive model.

Correlated	Sum of squared error (*Q*)	rms^a^ deviation (dev_rms_)	Sum of squared relative error (*Q*_*rel*⁡_)	Sum of relative error (*δ*)	Correlation coefficient (*γ*)	Range analysis: predicted data points within specified range of experimental results			
						±15%	±10%	±7.5%	±5%
Training sets									
Sphere	1.8282	0.1062	33.6267	27.854	0.4843	75.93	59.88	47.53	30.25
Cylinder	0.119	0.0251	0.7264	7.093	0.974	97.36	95.24	87.3	76.72
Rectangular prism	0.0507	0.0386	0.3259	2.1079	0.9792	91.18	85.29	79.41	61.77
All particles	1.9978	0.072	34.679	37.0549	0.8773	87.79	79.48	69.87	55.84
Test sets									
Sphere	0.9038	0.1294	19.2326	11.7315	0.4675	72.22	66.67	51.85	35.19
Cylinder	0.0402	0.0255	0.2236	2.5431	0.9669	96.77	90.32	83.87	72.58
Rectangular prism	0.01124	0.0306	0.0634	0.6657	0.7792	91.67	91.67	83.33	58.33
All particles	0.9552	0.08639	19.5205	14.9403	0.7804	85.94	80.47	70.31	55.47

^a^rms indicates root-mean square.
